# The stock market reaction to COVID-19 vaccination in ASEAN

**DOI:** 10.12688/f1000research.110341.2

**Published:** 2023-04-25

**Authors:** Marizsa Herlina, Ade Yunita Mafruhat, Eti Kurniati, Wildan Wildan, Hilwa Gifty Salsabila

**Affiliations:** 1 Department of Statistics, Universitas Islam Bandung, Bandung, 40116, Indonesia; 2 Department of Economic Development, Universitas Islam Bandung, Bandung, 40116, Indonesia; 3 Department of Mathematics, Universitas Islam Bandung, Bandung, 40116, Indonesia

**Keywords:** Stock reaction, vaccination, ASEAN, panel regression, heterogeneity, cross-dependence

## Abstract

Previous studies have shown that the confirmed cases drive investor sentiment, reflecting the stock's return. Based on this, the vaccination growth is also expected to drive the investor’s sentiment, which can be reflected in the return of the stock market in ASEAN.  Therefore, this study explores the vaccination impact on stock returns in ASEAN countries. This study contributes to the gap of taking the COVID-19 vaccination impact to the stock return into account by using the panel regression model with HC and Driscoll and Kraay robust covariance matrix estimator, which addresses the cross-dependency and heterogeneity problems. This study is one of the early studies of the topic, especially in ASEAN. The panel regression model with HC and Driscoll and Kraay robust covariance matrix estimator uses three variables: the daily stocks return, vaccine growth, and cases growth. It is a balanced panel data that includes six countries and 117 daily series data, making 702 observations used in the study.  The results show conflicting results where daily vaccination growth negatively affects the stock return. This problem can arise for several reasons, such as the uncertainty in the financial market and cross-dependency and heterogeneity detected in the model. We can see that the investors still have a negative sentiment because COVID-19 has resulted in uncertainty on the financial market in ASEAN. This gives us practical implications that the ASEAN country members’ government needs to push vaccination policy more aggressively.

## Introduction

No one could predict that the COVID-19 pandemic would be a long-lasting problem facing most of the world. All industries are suffering from the ongoing COVID-19 pandemic, until recently. This was shown from the estimation of the world’s gross output (GO) in 2020, which was −3.5; specifically, all countries also had a negative GO. For Association of Southeast Asian Nations (ASEAN), the economic growth prospect is quite dark due to their strong dependencies on the tourism sector. This sector provided 12% of ASEAN GDP in 2016, ^1^ and it was predicted to rise had there been no pandemic. The pandemic would make it hard to recover the tourism sector because of the travel limitation and quarantine policies applied differently for each country’s borders. ^2^ In the third quartal of 2020, most ASEAN countries suffered from the decrease of GDP except for Vietnam, which increased 2.91% of their GDP. ^3^ While pandemics are still ongoing, ASEAN will face a significant hurdle in its tourism sector, and policymakers should make a new strategy to increase economic growth.

One of the efforts to overcome the rising cases of COVID-19 is the vaccination program. Since the first quarter of 2021, almost all countries have started a COVID-19 vaccination program; however, the impact of the COVID-19 vaccination on economic growth is still rarely explored because the program is still in the early stages and there are not enough data to analyze. Despite that, the vaccination program is indeed an excellent start to cope with the spread of COVID-19. The program’s outcome is to slow down the spread and reduce positive cases of the COVID-19. The increasing number of vaccinated people can stabilize a saturated condition. With the growing number of people vaccinated, people will feel more secure and think positively about the future, which will help raise a reasonable expectation in the stock market.

Moreover, research in Vietnam ^4^ about the COVID-19 impact on the stock market shows that when Vietnam announced 0 cases of COVID-19 after the lockdown, the stock market in Vietnam raised significantly and became the best performing stock in April and May 2020. Based on this, the impact of the vaccination program is expected to have a similar outcome which is the increase of the stock market performance in other countries. However, it is not always the case that the positive sentiment will always increase the market’s return; otherwise, it can bring down the return because of the abnormal trading volume in the market. ^5^ Especially in a period of uncertainty, the investor’s behavior is hard to predict, reflecting on the stock returns.

Every country has different conditions and policies; this implies a variety of vaccination impacts to their stock market for each country. Therefore, panel data analysis can be used to see the stock reaction to the vaccination program in ASEAN. Panel data can control individual heterogeneity and identify the impact better than pure cross-section or time-series data only. ^6^^,^^7^ Panel data analysis is often used in analyzing the stock market responses study from the COVID-19 outbreak. ^8^^–^^11^ They used the stock return to see the market reaction and positive confirmed cases in the respective countries.

However, the Ordinary Least Square (OLS) estimation of the panel regression model requires the assumption of normally distributed and homogenous errors, which are rarely met in many cases in real-life data, especially economic data such as stock prices. If the assumption of homogeneity is violated, the estimator will be biased. Many previous studies used OLS estimation but did not mention diagnostic testing. Hence, we address the issue of where heterogeneity and cross-dependency occur in the panel model error. Therefore, the Heteroskedasticity consistent (HC) estimator can be an alternative estimator to have robustness in heterogeneity. However, heterogeneity can also exist from cross-dependency, and Driscoll and Kraay’s robust covariance matrix estimator addresses those problems and improve the model results for vaccination on stocks’ return. Several researchers had studied the financial market performance response to the COVID-19 vaccination. ^12^^–^^14^ These studies show that vaccination gives a significant effect on the global market. However, the variety of vaccines and each country’s vaccination policies would result in different stock market reactions. Besides, no study considers the vaccination impact in ASEAN countries, to the best of our knowledge.

Based on the previous studies, this study has three significant contributions. First, this study could picture the impact of vaccination on stock returns, and it also shows the benefit or the loss for the future investor. Second, the object of this study is the six biggest ASEAN country members: Indonesia, Thailand, Philipines, Vietnam, Singapore, and Malaysia. The 6 countries are the highest GDP countries in ASEAN, so We consider these countries to significantly influence the ASEAN stock market. ^15^ The study’s outcome is expected to help the ASEAN policymakers create a policy that considers the vaccination effect on ASEAN stock returns. Third, this study detected several problems, such as cross-dependency and heterogeneity, potentially leading to biased testing results. Thus, this study used the HC and Driscoll and Kraay robust covariance matrix estimator to address those problems and improve the model results for vaccination on stocks’ return. This paper then explains the whole research process, and it is divided into several sections: introduction, background and literature review, data and methodology, results, discussion, and conclusion.

## Background and literature review

The COVID-19 pandemic affects the stock market. ^4^ In the USA, COVID-19 impacted the US stock market volatility more than any other pandemic since the 1900s. ^16^ The stock market itself is strongly interconnected. If we learned from the previous pandemic, such as swine flu, the link from influenza to the stock market could be seen when it began to affect the key individuals massively in the stock market, such as traders, market makers, and overall investing behavior directly or indirectly via decreasing in liquidity due to the decline in information flows and production. ^17^ On the other hand, the pandemic can also negatively change investors' sentiment, which will affect the investment decision where it will be reflected in the stock prices. ^18^ In addition, research involving 64 countries ^19^ showed a negative impact of the COVID-19 pandemic on the stock return. In other words, stock prices reflect the investor’s expectations. When the downfall of the stock market can be seen as a pause in economic activities, it also means that there is price pressure from people’s expectations and fear of the investor’s. ^10^ The research conducted in India about the stock market reaction before and after lockdown showed that the stock return reacted positively to the policy after lockdown announcement. ^20^ The vaccination program and policies may also impact the stock market based on that knowledge.

The COVID-19 vaccination started on February 18 ^th^, 2021, where the high-risk population is the vaccination’s priority target. ^21^ More than 905 million vaccine doses have already been registered worldwide, which means there are 12 doses available for every 100 people. But, the gap between the available dose and the world population still exists. ^22^

**Figure 1.**
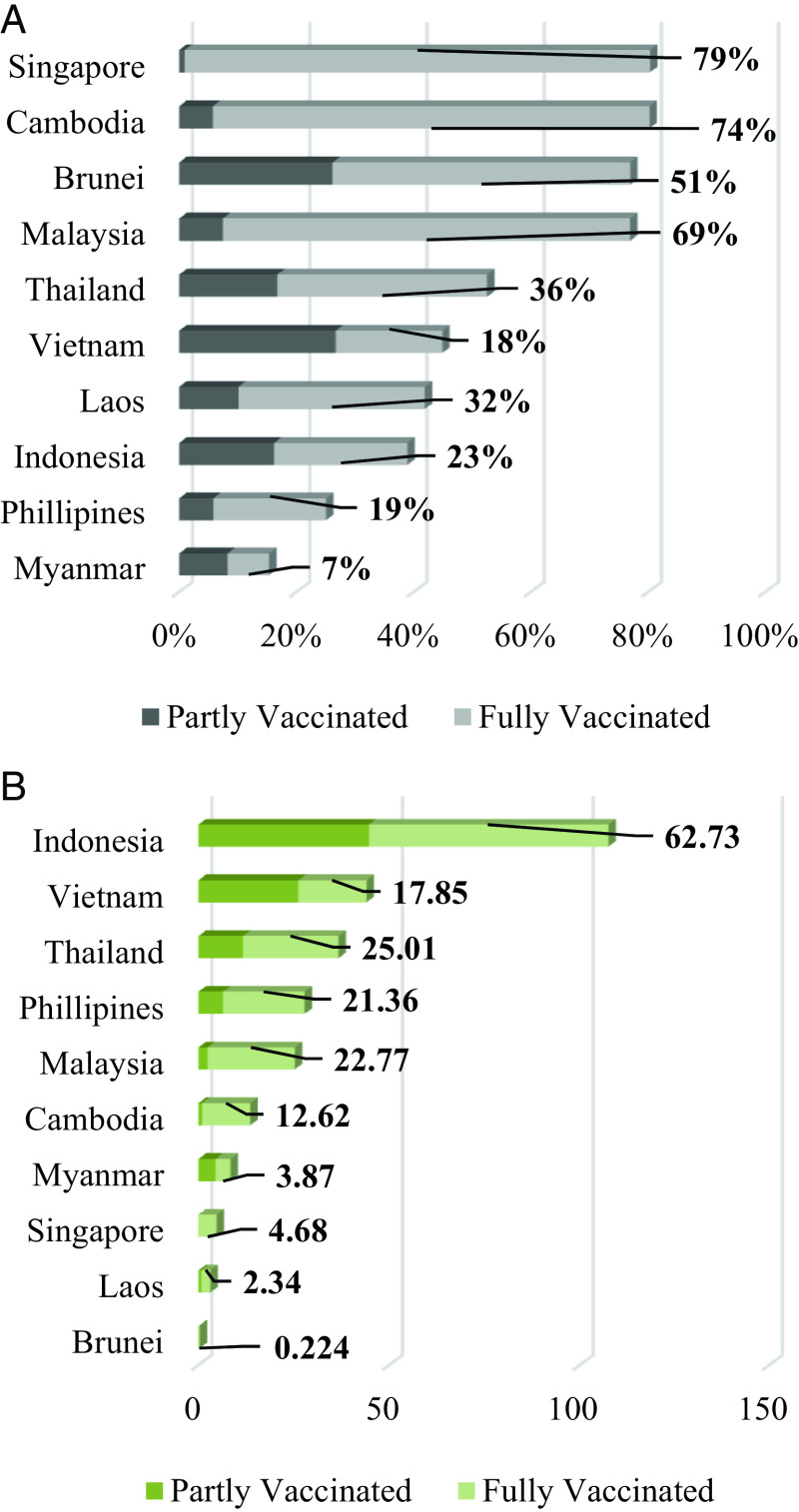
The percentage (A) and total number (in a million people) of the COVID-19 vaccinated people (B) in ASEAN countries on October 17 ^th^, 2021.

The vaccination program in ASEAN has begun differently in its member countries. The earliest country to start the vaccination program was Indonesia on January 26 ^th^, 2021. From Figure 1A, we can see that the three highest vaccinated countries in ASEAN are Singapore, Cambodia, and Brunei, while the lowest three are Indonesia, Philipines, and Myanmar. We also need to consider the size of the population in this case; in terms of the number of vaccinated people, Indonesia, Vietnam, and Thailand are the three highest countries in ASEAN ( Figure 1B). This shows that ASEAN countries have been conducting the vaccination program, and this study explored the stock market reaction to the vaccination program in ASEAN countries using panel data analysis.

The panel data analysis has been used often in analyzing the stock market reaction. The research by ^8^ used the panel regression to see the effect of uncertainty and confirmed cases on the stock market returns in 43 countries; he found that the higher confirmed cases impact greater with the country which also has a higher level of uncertainty. Similar studies were also done by, ^9^ who studied 47 countries on the panel regression to explore the effect of people’s trust in the government and society and also the confirmed cases to the stock market volatility. They found that trust in government and society is significantly crucial to market volatility.

The stock market reaction study in G-20 countries by ^10^ also used panel data regression and event study to see the impact of the COVID-19 outbreak on the abnormal returns of the stock and found that the COVID-19 outbreak has a negative effect to the stock returns. Moreover, the research of, ^11^ who studied the impact of freedom and growth rate of COVID-19 daily returns, also used a panel regression. He found that the growth of COVID-19 significantly affected the returns negatively, and there is a strong negative relationship between the country’s freedom and the effect of the pandemic in the stock market.

The link between the vaccine program and the stock market is also mentioned in several studies. The study about the response of the global stock market to vaccine availability had been explored by using five main markets indexes such as Dow Jones, Shanghai, S&P, FTSE, and EURONEXT). The results showed that after the vaccine arrival, the stock prices significantly outweighed before the vaccine arrived. ^12^ Another study using the panel data model of the volatility of the stock market reaction to the vaccination program in the international financial market shows that the stock market volatility significantly dropped by the mass vaccinations. ^13^ The vaccine effectivity on the stock market was analyzed using a wavelet coherence approach in the USA. The COVID-19 vaccination, infection rate, and the case fatality ratio significantly influence the S&P-500 returns at the majority business cycle. In addition, a recent study shows that news about covid help equities in general, whether its positive or negative news. ^23^ Moreover, the Covid-19 vaccine announcement also positively impacted the Chinese stock market. Besides, many studies prove that mass campaign about Covid-19 vaccines receives positive sentiments from investors. ^23^^–^^26^ Therefore, the approvals of Covid-19 vaccines lighted the hope of humanity and economic recovery, and this phenomenon is reflected in the stock market. ^14^^,^^25^ However, none of them consider the existence of cross-dependency and heterogeneity problems. Therefore, this study incorporated the cases and vaccine of COVID-19 growth to the ASEAN stock returns. Because the COVID-19 vaccine growth rate and positive cases are unrelated, the panel model regression will be used, considering there is no endogeneity in the variables used. We also used the HC estimators to address the issue of heterogeneity and Driscoll and Kraay robust covariance matrix estimator to manage the cross-dependency in the panel data model.

## Methods

### Ethics

This study used secondary datasets obtained from investing.com and ^27^ that are available online. Thus, there are no ethical issues in this study.

### Data

The variable used in this study are the daily stock returns, ^10^ which use equation 1:

$$R_{\mathrm{it}}=\ln\left[\frac{P_{i,t}}{P_{i,t-1}}\right]\times 100$$ (1)

Where $R_{\mathrm{it}}$ is the daily return for index i and $P_{i,t}$ is the closing stock price of the i index stock at the time t and $P_{i,t-1}$ is the closing stock price of the *i* index stock at the time *t*−1 (a day before). The return was calculated from the available stock indexes in ASEAN ( Table 1) available (among the ASEAN members, only six countries had a country stock index) from investing.com. The other variables are the growth of the confirmed positive COVID-19 cases and the growth of the vaccinated people, which follows equations 2 and 3. ^9^ All data are secondary data, originated in the form of total vaccination and confirmed cases of COVID-19 that are gathered from ^27^ and are accessed on July 8 ^th^, 2021. All of the analyses on this paper were done using the R software.

$${\text{Case Growth}}_{i}=\ln\left[\frac{{\text{Case}}_{i,t}}{{\text{Case}}_{i,t-1}}\right]$$ (2)

$${\text{Vaccine Growth}}_{i}=\ln\left[\frac{{\text{Vaccinated}}_{i,t}}{{\text{Vaccinated}}_{i,t-1}}\right]$$ (3)

where ${\text{Case Growth}}_{i,t}$ is the confirmed case at the *i* country at period *t* and ${\text{Vaccine Growth}}_{i,t}$ is the amount of the vaccinated people in the *i* country at period *t*. All of the variables are available in daily series, the period of this study is when the ASEAN country have already started their vaccination program from March 13 ^th^, 2021, until July 7 ^th^, 2021.

**Table 1.** The stock index list in ASEAN members.

No	ASEAN members	Stock index
1	Indonesia	IHSG or JCI
2	Thailand	SET Index
3	Phillipines	PSE Index
4	Vietnam	VN Index
5	Singapore	Straits Times Index
6	Malaysia	FTSE Bursa Malaysia Index

### Methodology

The general model, ^28^ which are estimated in the panel regression and the variables used follow equation 4:

$$R_{\mathrm{it}}=x_{\mathrm{it}}\beta +c_{i}+\varepsilon_{\mathrm{it}}$$ (4)

Where $x_{\mathrm{it}}=\left[\ln{\left(\text{Cases Growth}\right)}_{i,t},\ln{\left(\text{Vaccine Growth}\right)}_{i,t}\right]\prime$ ; $c_{i}=z\prime_{i}\alpha$ is the individual effect which contains group-specific variables. The models consist of ^28^^,^^29^:

1. Pooled Model which follows the equation 5.
$$R_{\mathrm{it}}=x_{\mathrm{it}}\beta +v_{\mathrm{it}}$$ (5)
  where $v_{\mathrm{it}}\equiv c_{i}+u_{\mathrm{it}}$ as the composite errors; $c_{i}$ is individual effect; $u_{\mathrm{it}}$ is the idiosyncratic errors and t=1, …, T.
2. Fixed Effect Model which follows the equation 6.
$$R_{\mathrm{it}}=x_{\mathrm{it}}\beta +c_{i}+\varepsilon_{\mathrm{it}}$$ (6)
  where $c_{i}=z\prime_{i}\alpha$ and $\varepsilon_{\mathrm{it}}$ is an error term.
3. Random Effect Model which follows the equation 7. ^30^
$$R_{\mathrm{it}}=x_{\mathrm{it}}\beta +\alpha +v_{\mathrm{it}}$$ (7)
  where $v_{\mathrm{it}}=c_{i}j_{T}+u_{i}$ ; $j_{T}$ is the Tx1 vector of ones and $u_{i}$ is a group-spesific random element (for each country). Then, after these models are estimated, several testings were carried out in order to see which model perform best such as i) The Hausman test ^31^ is used for determining the most suitable model between fixed effect and random effect model which the null hypothesis is Random Effect Model is more suitable for the data; ii) The Breusch-Pagan Lagrange Multiplier Test ^32^ in order to see the cross-sectional dependency. In the event of cross-dependency exist, the Driscoll and Kraay robust covariance matrix estimator will be used ^33^; iii) The Breusch-Pagan Test ^34^ in order to see the homoskedasticity assumption. If there is any heteroskedasticity detected, the heteroskedasticity consistent (HC) estimation can be used for the model estimation. ^30^^,^^35^^,^^36^

## Results

### Descriptive analysis

**Figure 2.**
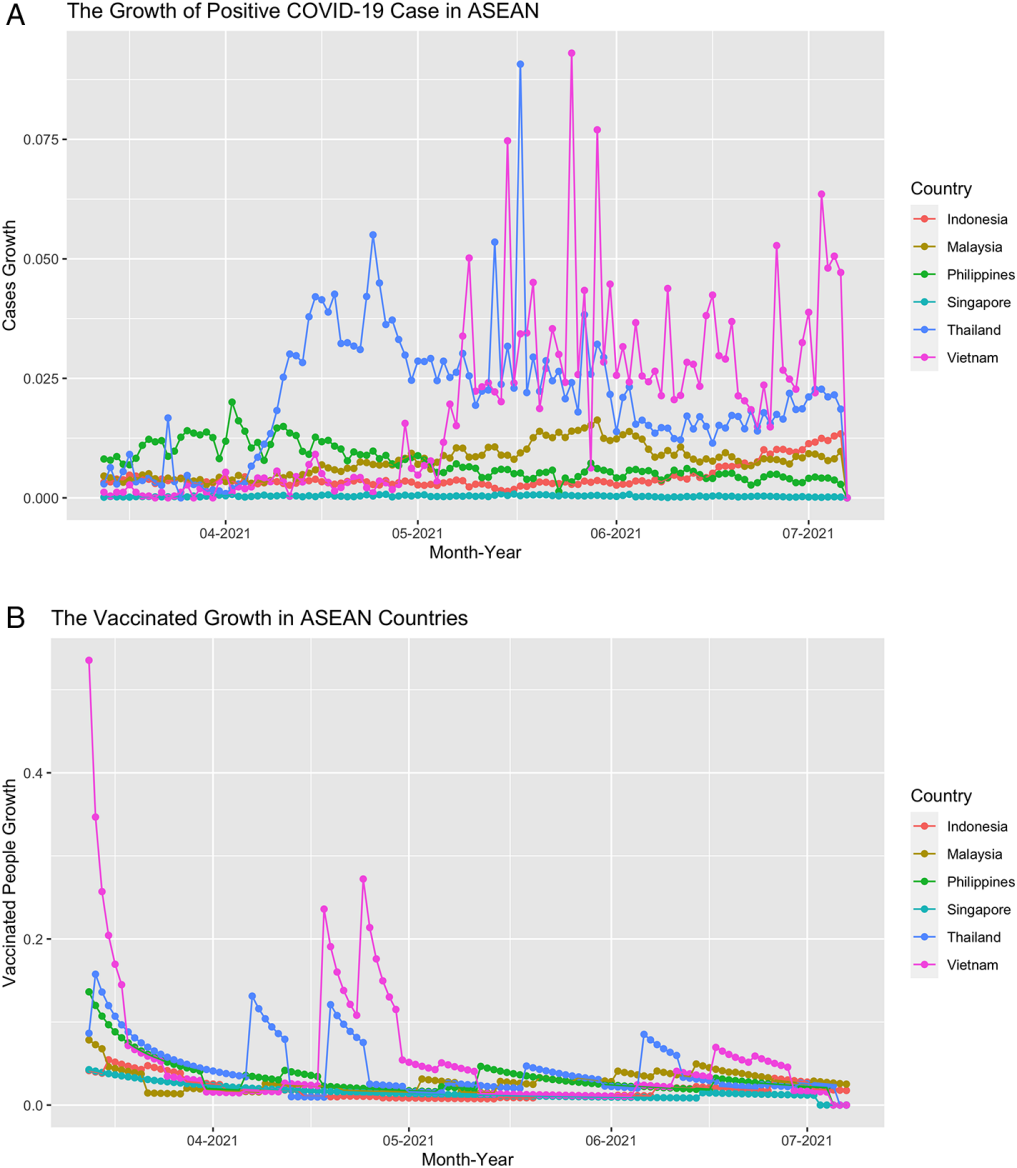
The positive case growth of COVID-19 (A) and the vaccinated people growth (B) in ASEAN countries from March 13 ^th^, 2021 until July 7 ^th^, 2021.

**Figure 3.**
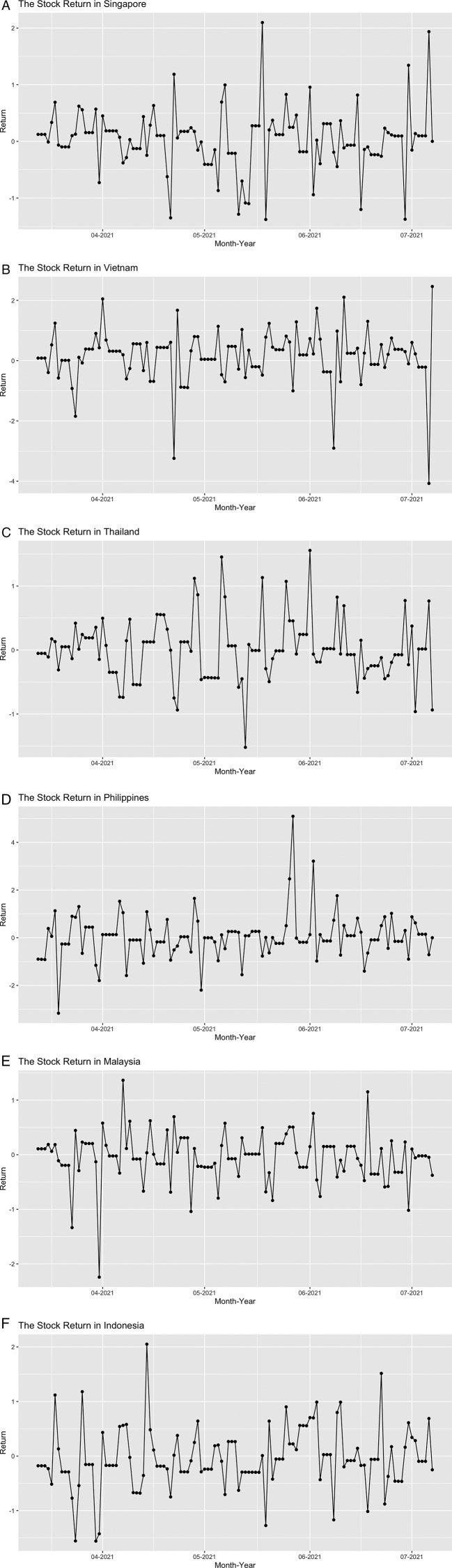
The daily stock index return in Singapore (A), Vietnam (B), Thailand (C), Philippines (D), Malaysia (E), and Indonesia (F) from March 11 ^th^, 2021 until July 7 ^th^, 2021.

This study used balanced panel data which includes six countries and 117 daily series so the total is 702 observations. From Figure 2, we can see that the growth of confirmed cases and the vaccinated people fluctuate over time. For confirmed cases growth, it fluctuates mostly in Vietnam, Thailand, Malaysia, and the Philippines. Meanwhile, the vaccination growth decreased over time and sometimes it stays flat because the growth did not change that much. If we see the series on Figure 3, the movement of each country evolved around the 0 line, which confirms that the mean of the daily stock return is near 0.

### Panel regression model of the stock reaction

The model estimation for Pooled Model, Fixed Effect Model, and Random Effect Models are provided in Table 2. From the estimates, vaccine growth is the only one that significantly affects the return of the stock in ASEAN countries. Moreover, both pooled and random effect models produce similar results in the coefficient. All of the estimations for vaccine growth have negative signs, which means that the vaccine growth negatively influences the return of the stock, which is in contrast to the expectation.

**Table 2.** Estimation results of panel data regression.

Variables	Statistics	Pooled estimation	Fixed effect estimation	Random effect estimation
Vaccine growth	Coefficient	-0.546	-1.365	-0.546
	Std. Error	0.664	0.742	0.664
	p-value	0.411	0.066 *	0.411
Cases growth	Coefficient	-0.002	-3.534	-0.002
	Std. Error	2.102	2.749	2.102
	p-value	0.99	0.199	0.99
Constant	Coefficient	0.038	-	0.038
	Std. Error	0.039	-	0.039
	p-value	0.332	-	0.332
Model summary	Observations	702	702	702
	R ^2^	0.001	0.006	0.001
	Adjusted R ^2^	-0.002	-0.004	-0.002
	F-Statistic	0.3374	1.99723	-

Notes: Rejecting the null hypotheses at

* 10%;

** 5%;

*** 1% significance level.

After the model estimation, several tests are conducted to see the most suitable model among all using the Hausman test and Breusch-Pagan Lagrange Multiplier test ( Table 3). The Hausman test shows that the fixed effect model is more ideal than the fixed random effect model, which means there is heterogeneity in vaccine growth and case growth on a daily basis. But then the Breusch-Pagan Lagrange Multiplier test shows that there is a cross-sectional dependence problem. This means that there is a dependence on the stock returns among ASEAN countries.

**Table 3.** The diagnostic testing of the models.

Test	Statistics value	P-value
Hausman Test	10.812	0.0045 ***
Breusch-Pagan Lagrange Multiplier Test	65.237	0.000 ***
Breusch-Pagan Test	127.41	0.00 ***

Notes: Rejecting the null hypotheses at

* 10%;

** 5%;

*** 1% significance level.

In addition, the Breusch-Pagan test also shows that the variance is not homoskedastic so we estimate the HC estimators for the fixed-effect model which are robust to heteroskedasticity and Driscoll and Kraay robust covariance matrix estimator for cross-dependency problem in Table 4. The HC estimators and the Driscoll and Kraay robust covariance matrix estimators of the fixed effect model shows different results to the previous estimations in Table 2 in terms of the standard error and the p-value. For the HC estimators, all variables significantly affect the daily return of the stock in the ASEAN country. The cases growth indeed negatively influences the return of the stock significantly but the vaccine growth also has a negative coefficient. But, if we use the Driscoll and Kraay robust covariance matrix estimators, all of the variables do not significantly influence the return. The HC and Driscoll and Kraay Robust Covariance Matrix Estimators have different results in testing. Even though we have already addressed each problem, they still have biased results.

**Table 4.** The estimation of fixed effects model by using HC estimators and Driscoll and Kraay Robust covariance matrix estimator.

Variables	HC estimators			
	Estimate	Std. Error	T-value	P-value
Vaccine growth	-1.365	0.496	-2.7515	0.006 ***
Cases growth	-3.534	1.348	-2.6218	0.009 ***

Variables	Driscoll and Kraay Robust covariance matrix estimators			
	Estimate	Std. Error	T-value	P-value
Vaccine growth	-1.365	0.864	-1.579	0.1147
Cases growth	-3.534	3.212	-1.1	0.2717

Notes: Rejecting the null hypotheses at

* 10%;

** 5%;

*** 1% significance level.

## Discussion

Based on the results above, it is shown that the cases growth negatively impacted the stock returns in ASEAN. This is in line with all of the previous studies. ^8^^,^^9^^,^^11^ But we found conflicting results in the vaccination growth. The vaccination growth is supposed to impact the stock return positively, but it harms the stock return. This is in line with the theory that the positive sentiment does not necessarily raise the stock returns. ^5^^,^^13^ This proves that many things happen in trading. They do not move solely on investors’ sentiment, but many factors interfere with the market, such as government interventions, news, abnormal trading, *etc.* The goal of the vaccination is herd community which makes a certain proportion of the population have the immunity of a disease. Until now, except for the USA, the world is still far behind the herd immunity threshold. ^37^ If the goal of herd immunity had not been achieved, then the stock market is still in a state of extreme uncertainty; that is why the investor behavior would be hard to predict. ^5^ Future investors must be aware of the risk if they want to invest in this situation. The practical implication of this study is that ASEAN countries need to create strategies that will outweigh the risk of this uncertainty to attract investors such as strengthening the healthcare system to ease the uncertainty. The more advanced countries’ financial markets are proven to be more robust to the pandemic effect because of their advanced technology, communication, and good citizen’s welfare. ^38^ The ASEAN countries should strengthen their citizens’ trust in them to stabilize the situation like The Phillippines did.

Second, the limitation in this study is the existence of either a cross-dependency problem or heterogeneity left in this model. The HC estimators only addressed the heterogeneity problem but not the cross-dependency problem. Reversely, the Driscoll and Kraay Robust Covariance Matrix Estimators is robust to the cross-dependency problem but not heterogeneity. Both cannot solve those problems simultaneously. The cross-dependency itself can be caused by spatial or spillover effect or unobserved common factors. ^39^ This means that every country has a dependency on each other. The spatial effect of the vaccination on the stock returns need to be explored more.

## Conclusions

This study explores the vaccination impact on stock returns in ASEAN by using panel regression. It is found that both vaccination growth and the growth cases are impacting the return of stocks in ASEAN. While the growth cases are in line with previous studies, there are conflicting results where the vaccination growth negatively affects the return stock in ASEAN. These results are contrasted with the expectation that vaccination should bring positive sentiment to the investors. The study confirms the research objectives that we found several mixed results in vaccination impact and address the problem of cross-dependency and heterogeneity. These mixed results could be because of the investor’s sentiment, which is in extreme uncertainty because of the non-presence of herd immunity until now. We can see that the investors still have a negative sentiment because COVID-19 has resulted in uncertainty on the financial market in ASEAN. This gives us practical implications that the ASEAN country members’ government needs to push vaccination policy more aggressively. Even though the results showed that the vaccination still negatively influences the stock returns, the vaccination growth has not shown the distribution of the vaccinated people. For example, in Indonesia, only 56,04% of the Indonesian population gets the second vaccination.

Meanwhile, the new variant of COVID-19 keeps evolving and making a new peak on confirmed cases in many countries, including ASEAN. Thus, the economic activities would also be halted if these are not carefully taken care of. So, the ASEAN countries must fasten their second vaccine distribution, and after that, they need to ensure their citizen get the third vaccine. This is a significant move to stabilize the investors’ trust that governments can warranty the citizen’s welfare. This would make the economy slowly recovers, and the investors’ positive sentiment will follow eventually, and next, the financial market can be stabilized in the end.

Second, there is a cross-dependency and heterogeneity problem in the model which can cause biased test results. Therefore, future studies suggest using another estimator or model to address heterogeneity and the cross-dependency problem simultaneously. For example, consider the spatial panel modeling or another model such as the heterogeneous panel data models with cross-sectional dependence ^40^ that can address this problem to avoid biased test results.

## Data availability

### Underlying data

Github: Data of The Stock Market Reaction to COVID-19 Vaccination in ASEAN. Dataset created for https://github.com/owid/covid-19-data/tree/master/public/data/vaccinations ^27^

Data are available under the terms of the Creative Commons Attribution 4.0 International license (CC-BY 4.0).
