# Supplementary material for: The stock market reaction to COVID-19 vaccination in ASEAN
Source: F1000Res. 2023 Apr 25;11:363. Originally published 2022 Mar 29. [Version 2] doi: 10.12688/f1000research.110341.2 (PMC10422054; doi:10.12688/f1000research.110341.2)
Supplement: Peer review history

# Peer review: Reviewer response for version 2

**DOI:** 10.5256/f1000research.146719.r186550

**Published:** 2023-08-11

**Authors:** Walid Bakry

**Affiliations:** 1 University of Western Sydney, Sydney, Australia

I checked the revisions round 1 required and I can see that author(s) did address all of the reviewers’ questions.

 The article is interesting, although the topic is not new, however, there are couple of points I would suggest the authors to consider:

 1 - The paper did not mention early studies which tested the impact of COVID-19 vaccine on stock return or return volatility, these should be mentioned and included in the literature review section. This includes:

- Apergis, N., Mustafa, G., & Malik, S. (2022). COVID-19 pandemic, stock returns, and volatility: the role of the vaccination program in Canada. *Applied Economics*, *54*(42), 4825–4838. https://doi.org/10.1080/00036846.2022.2036688
- Bakry, W, Kavalmthara, P, J, Saverimuttu V, Liu Y, Cyril S, (2022). Response of stock market volatility to COVID-19 announcements and stringency measures: A comparison of developed and emerging markets. *Finance Research Letters*, *46*, 102350–102350. https://doi.org/10.1016/j.frl.2021.102350
- Behera, J., Pasayat, A. K., & Behera, H. (2022). COVID-19 Vaccination Effect on Stock Market and Death Rate in India. *Asia-Pacific Financial Markets*, *29*(4), 651–673. https://doi.org/10.1007/s10690-022-09364-w
- Chan, K. F., Chen, Z., Wen, Y., & Xu, T. (2022). COVID-19 vaccines and global stock markets. *Finance Research Letters*, *47*, 102774–102774. https://doi.org/10.1016/j.frl.2022.102774

2- The second point is the source of stock indices data shown in table 1?

 3- The third and final point of concern for me in this article is the use of a panel model with only 2 independent variables which explain little about the change in stock returns. You need to add some other control variables, so you don’t fall in the omitted variable bias issue. That is why your R ^2^ is so low and you are not getting much significance in Table 2.

Is the work clearly and accurately presented and does it cite the current literature?

Partly

If applicable, is the statistical analysis and its interpretation appropriate?

Partly

Are all the source data underlying the results available to ensure full reproducibility?

Partly

Is the study design appropriate and is the work technically sound?

Partly

Are the conclusions drawn adequately supported by the results?

Yes

Are sufficient details of methods and analysis provided to allow replication by others?

Yes

Reviewer Expertise:

NA

I confirm that I have read this submission and believe that I have an appropriate level of expertise to confirm that it is of an acceptable scientific standard, however I have significant reservations, as outlined above.

## References

[1] : COVID-19 pandemic, stock returns, and volatility: the role of the vaccination program in Canada. *Applied Economics* .2022;54(42) : 10.1080/00036846.2022.2036688 4825-4838 10.1080/00036846.2022.2036688

[2] : Response of stock market volatility to COVID-19 announcements and stringency measures: A comparison of developed and emerging markets. *Financ Res Lett* .2022;46: 10.1016/j.frl.2021.102350 102350 10.1016/j.frl.2021.102350 35431683PMC8994447

[3] : COVID-19 Vaccination Effect on Stock Market and Death Rate in India. *Asia-Pacific Financial Markets* .2022;29(4) : 10.1007/s10690-022-09364-w 651-673 10.1007/s10690-022-09364-w

[4] : COVID-19 vaccines and global stock markets. *Financ Res Lett* .2022;47: 10.1016/j.frl.2022.102774 102774 10.1016/j.frl.2022.102774 35283695PMC8898767

# Peer review: Reviewer response for version 1

**DOI:** 10.5256/f1000research.121936.r139087

**Published:** 2022-05-30

**Authors:** Evan Lau

**Affiliations:** 1 Faculty of Economics and Business, Universiti Malaysia Sarawak, Kota Samarahan, Malaysia

1. It is a rather interesting proposition of linking the vaccination and stock market. The authors should be able to provide the theoretical link on how these two were related. At the same time, authors could explore using not just specifically COVID, but also pandemics themselves to find the link.

 2. Why the selected ASEAN countries? How do these 6 countries represent ASEAN?

 3. As the research on the topical issue is on-going, it might be a good idea to expand the sample period at least to 2022. With an expanded sample, I am sure there are more interesting findings. The authors could explore the sub-sample estimation based on the big sampling data.

 4. Driscoll and Kraay Robust Covariance Matrix Estimators - I do not see the relevance of adopting this method. Perhaps the authors could consider other methods or other panel estimates. A non-stationary panel time series perhaps?

 5. Low R2 and negative adjusted R2.

Is the work clearly and accurately presented and does it cite the current literature?

Yes

If applicable, is the statistical analysis and its interpretation appropriate?

Yes

Are all the source data underlying the results available to ensure full reproducibility?

Yes

Is the study design appropriate and is the work technically sound?

Yes

Are the conclusions drawn adequately supported by the results?

Yes

Are sufficient details of methods and analysis provided to allow replication by others?

Yes

Reviewer Expertise:

Economics

I confirm that I have read this submission and believe that I have an appropriate level of expertise to confirm that it is of an acceptable scientific standard, however I have significant reservations, as outlined above.

# Response

**Published:** 2023-04-07

**Authors:** Marizsa Herlina

Thank you for your suggestions. We have accommodated all of your requests in the revised papers as follows:

**1. Reviewer:** It is a rather interesting proposition of linking the vaccination and stock market. The authors should be able to provide the theoretical link to how these two were related. At the same time, authors could explore using not just specifically COVID, but also pandemics themselves to find the link.

**Answer**: when the previous pandemic hits such as swine flu or influenza, it has been found that it has link to the stock market when it hits massively, and this has been addressed in the 1st paragraph of the Background and literature review of the revised paper as follows:

 “If we learned from the previous pandemic, such as swine flu, the link from influenza to the stock market could be seen when it began to affect the key individuals massively in the stock market, such as traders, market makers, and overall investing behavior directly or indirectly via decreasing in liquidity due to the decline in information flows and production”

**2. Reviewer:**Why the selected ASEAN countries? How do these 6 countries represent ASEAN?

**Answer**: The 6 countries, Indonesia, Thailand, Vietnam, Philippines, Singapore, Malaysia are the highest GDP countries in ASEAN, so they significantly drive the ASEAN economies.

 We have accommodated this in the 6th paragraph of the revised paper.

 “Second, the object of this study is the six biggest ASEAN country members: Indonesia, Thailand, Philipines, Vietnam, Singapore, and Malaysia. The 6 countries are the highest GDP countries in ASEAN, so We consider these countries to significantly influence the ASEAN stock market (1).”

**3. Reviewer: **As the research on the topical issue is on-going, it might be a good idea to expand the sample period to at least 2022. With an expanded sample, I am sure there are more interesting findings. The authors could explore the sub-sample estimation based on the big sampling data.

**Answer**: Thank you for the suggestions. This paper limits the time window to 2021 because the sentiment mostly happens when the vaccine is launched. But we will consider your suggestions for future research.

**4.** **Reviewer: **Driscoll and Kraay Robust Covariance Matrix Estimators - I do not see the relevance of adopting this method. Perhaps the authors could consider other methods or other panel estimates. A non-stationary panel time series perhaps?

**Answer**: The Driscoll and Kraay Robust Covariance Matrix Estimators can estimate the model when there is heterogeneity from cross-dependency detected, and it is already stated in the paper result section in the last paragraph:

 “In addition, the Breusch-Pagan test also shows that the variance is not homoskedastic so we estimate the HC estimators for the fixed-effect model, which are robust to heteroskedasticity and Driscoll and Kraay robust covariance matrix in Table 4.”

 And in the methodology, last paragraph:

 “In the event of cross-dependency exist, the Driscoll and Kraay robust covariance matrix estimator will be used; iii) The Breusch-Pagan Test in order to see the homoskedasticity assumption. If there is any heteroskedasticity detected, the heteroskedasticity consistent (HC) estimation can be used for the model estimation.”

 And also in the 5 ^th^ paragraph on the revised paper we have added more explanation about this:

 “However, the Ordinary Least Square (OLS) estimation of the panel regression model requires the assumption of normally distributed and homogenous errors, which are rarely met in many cases in real-life data, especially economic data such as stock prices. If the assumption of homogeneity is violated, the estimator will be biased. Many previous studies used OLS estimation but did not mention diagnostic testing. Hence, we address the issue of where heterogeneity and cross-dependency occur in the panel model error. Therefore, the Heteroskedasticity consistent (HC) estimator can be an alternative estimator to have robustness in heterogeneity. However, heterogeneity can also exist from cross-dependency, and Driscoll and Kraay's robust covariance matrix estimator addresses those problems and improve the model results for vaccination on stocks’ return.”

 A non-stationary panel time series such as VECM panel is not used due to the assumption that both vaccination rate and case growth are independent, so the model has no endogeneity or multicollinearity. Hence, we used the panel data regression model. This has been addressed in the last paragraph of the Background and literature review as follows:

 “Because the COVID-19 vaccine growth rate and positive cases are unrelated, the panel model regression will be used, considering there is no endogeneity in the variables used.”

**5.** **Reviewer:** Low R2 and negative adjusted R2.

**Answer**: The low R2 mainly causes the negative adjusted R2, which means most of the dependent variability was not explained by the independent variables. So it translates that the vaccination rate and case growth do not explain most of the variability of the stock prices in the initial panel model. And there is also a lack of assumption fulfilments in heterogeneity and cross-dependency in the initial panel model, so the next step is we estimate the model again using the HC estimators for the fixed-effect model, which are robust to heteroskedasticity and Driscoll and Kraay robust covariance matrix estimator for the cross-dependency problem. (This has been addressed in the previous question).

# Peer review: Reviewer response for version 1

**DOI:** 10.5256/f1000research.121936.r134887

**Published:** 2022-05-04

**Authors:** Robiyanto Robiyanto

**Affiliations:** 1 Faculty of Economics and Business, Satya Wacana Christian University, Salatiga, Indonesia

Good manuscript, however some improvements should be made prior to indexing. This manuscript can contribute to the reference regarding the dynamics of financial markets in the pandemic era. Minor changes required.

**Major points:**

- Please explain how vaccination could affect stock return? As you know, some stock markets plummeted in the early pandemic event, but then these stock markets then recovered because the pandemic was priced in already. This is not because of merely vaccination, some robustness checking is needed. The awakening of retail investors (i.e. in Indonesia) etc. should be considered.

**Minor points:**

- Please sharpen the research gaps, which need your contributions, especially the third contribution. Please describe some flaws in previous studies which need this solution and how your method could eliminate these flaws.
- Add some recent literature. Some related literature has been published.

Is the work clearly and accurately presented and does it cite the current literature?

Yes

If applicable, is the statistical analysis and its interpretation appropriate?

Yes

Are all the source data underlying the results available to ensure full reproducibility?

Yes

Is the study design appropriate and is the work technically sound?

Yes

Are the conclusions drawn adequately supported by the results?

Yes

Are sufficient details of methods and analysis provided to allow replication by others?

Yes

Reviewer Expertise:

NA

I confirm that I have read this submission and believe that I have an appropriate level of expertise to confirm that it is of an acceptable scientific standard.

# Response

**Published:** 2023-04-07

**Authors:** Marizsa Herlina

Thank you for your suggestions. We have accommodated all of your requests in the revised papers as follows:

**1. Major points:**

- **Reviewer: **Please explain how vaccination could affect stock return? As you know, some stock markets plummeted in the early pandemic event, but then these stock markets then recovered because the pandemic was priced in already. This is not because of merely vaccination, some robustness checking is needed. The awakening of retail investors (i.e. in Indonesia) etc. should be considered.

**Answer**: In recent studies, the link between vaccination and stock mostly happens in the news about vaccine announcements, resulting in positive sentiment by investors. We already added this in the revised paper in the 1st paragraph in the background and literature review as follows:

 “In addition, a recent study shows that news about covid help equities in general, whether its positive or negative news(3). Moreover, the Covid-19 vaccine announcement also positively impacted the Chinese stock market. Besides, many studies prove that mass campaign about Covid-19 vaccines receives positive sentiments from investors (3–6). Therefore, the approvals of Covid-19 vaccines lighted the hope of humanity and economic recovery, and this phenomenon is reflected in the stock market (5),”

**2. Minor points:**

- **Reviewer: **Please sharpen the research gaps, which need your contributions, especially the third contribution. Please describe some flaws in previous studies which need this solution and how your method could eliminate these flaws.

**Answer**: This has been addressed in the fifth paragraph of the Introduction in the revised paper as follows:

 "However, the Ordinary Least Square (OLS) estimation of the panel regression model requires the assumption of normally distributed and homogenous errors, which are rarely met in many cases in real-life data, especially economic data such as stock prices. If the assumption of homogeneity is violated, the estimator will be biased. Many previous studies used OLS estimation but did not mention diagnostic testing. Hence, we address the issue of where heterogeneity and cross-dependency occur in the panel model error. Therefore, the Heteroskedasticity consistent (HC) estimator can be an alternative estimator to have robustness in heterogeneity. However, heterogeneity can also exist from cross-dependency, and Driscoll and Kraay's robust covariance matrix estimator addresses those problems and improve the model results for vaccination on stocks’ return."

 And in the Background and literature review section, in the last paragraph:

 “However, none of them consider the existence of cross-dependency and heterogeneity problems. Therefore, this study incorporated the cases and vaccine of COVID-19 growth to the ASEAN stock returns.”

- **Reviewer: **Add some recent literature. Some related literature has been published.

**Answer**: We have added recent literatures as you can see in the first point and also from the other reviewer as follows:

 1. Yiu MS, Tsang A. Impact of COVID-19 on ASEAN5 stock markets. J Asia Pac Econ. 2021 Aug 21;1–14.

 3. Arteaga-Garavito MJ, Croce MM, Farroni P, Wolfskeil I. When the Markets Get CO.V.I.D: Contagion, Viruses, and Information Diffusion. SSRN. 2022;

 4. Hartono, Hartono. Covid-19 Vaccine: Global Stock Market “Game Changer.” J Asian Multicult Res Econ Manag Study [Internet]. 2021;2(2). Available from: https://amrsjournals.com/index.php/jamrems/article/view/102

 5. Ho KC, Gao Y, Gu Q, Yang D. Covid-19 vaccine approvals and stock market returns: The case of Chinese stocks. Econ Lett. 2022 Jun;215:110466.

 6. Oanh TTK. The impact of COVID-19 vaccination on stock market: is there any difference between developed and developing countries? Heliyon [Internet]. 2022 [cited 2023 Apr 2];8(9). Available from: https://linkinghub.elsevier.com/retrieve/pii/S2405844022020060
